# Supplementary material for: Strontium Treatment Potentiates Bone Anabolic Action of Intermittent PTH in Ovariectomized Rats
Source: Calcif Tissue Int. 2026 Jul 28;117(1):124. doi: 10.1007/s00223-026-01575-x (PMC13415679; doi:10.1007/s00223-026-01575-x)
Supplement: Supplementary file 1 — Supplementary Material 1 [file 223_2026_1575_MOESM1_ESM.docx]

**Supplementary Table 1**. List of primers used in real-time PCR.

| **Gene** | **Primer** | **Sequence** |
| --- | --- | --- |
| *B2m* | F | CACTGACCGGCCTGTATGCT |
|  | R | GTATGTTCGGCTTCCCATTCTC |
| *Rankl* | F | GCACACCTCACCATCAATGC |
|  | R | AGCCTCGATCGTGGTACCAA |
| *Opg* | F | GACAACGTGTGTTCCGGAAA |
|  | R | GGTAGGAACAGCAAACCTGAAGA |
| *Col1a1* | F | CTGGCCTTGGAGGAAACTTT |
|  | R | GCACGGAAACTCCAGCTGAT |
| *Alpl* | F | AGATGGCCTGGATCTCATCAGT |
|  | R | GTTCAGTGCGGTTCCAGACATA |
| *Bglap1* | F | GGAGGGCAATAAGGTAGTGAACAG |
|  | R | CACAAGCAGGGTTAAGCTCACA |
| *Igf1* | F | GCTGGTGGATGCTCTTCAGTT |
|  | R | CGAATGCTGGAGCCATAGC |
